# Telemedicine in Primary Health: The Virtual Doctor Project Zambia

**DOI:** 10.1186/1747-5341-6-9

**Published:** 2011-05-13

**Authors:** Evans N Mupela, Paul Mustarde, Huw LC Jones

**Affiliations:** 1 Walter Sisulu University, NMD Campus, Nelson Mandela Drive, Mthatha, RSA; 2 UNU-MERIT, Netherlands; 3 The Virtual Doctor Project, Virtual Development CIC, P.O Box 171, Lewes, East UK

## Abstract

This paper is a commentary on a project application of telemedicine to alleviate primary health care problems in Lundazi district in the Eastern province of Zambia. The project dubbed 'The Virtual Doctor Project' will use hard body vehicles fitted with satellite communication devices and modern medical equipment to deliver primary health care services to some of the neediest areas of the country. The relevance and importance of the project lies in the fact that these areas are hard-to-reach due to rugged natural terrain and have very limited telecommunications infrastructure. The lack of these and other basic services makes it difficult for medical personnel to settle in these areas, which leads to an acute shortage of medical personnel. We comment on this problem and how it is addressed by 'The Virtual Doctor Project', emphasizing that while the telemedicine concept is not new in sub-Saharan Africa, the combination of mobility and connectivity to service a number of villages 'on the go' is an important variation in the shift back to the 1978 Alma Ata principles of the United Nations World Health Organization [WHO].

This overview of the Virtual Doctor Project in Zambia provides insight into both the potential for ICT, and the problems and limitations that any "real-world" articulation of this technology must confront.

## Introduction

The rapid spread of the use of computers and communication technologies^i^, now commonly referred to as Information and Communication Technologies [ICT], in the last two decades has led to a plethora of ideas of applications for the benefit of poor people in remote areas of poor nations. One of the most important questions on the use of ICT has been the impact that these technologies can have on health service delivery to hard-to-reach areas in developing countries. This is because the central advantage and power of ICT is the inherent ability to deliver diverse information across large geographical spaces in relatively short time periods. Health practitioners in developing countries have seen this possibility and come up with useful ways of tapping the power of ICT to reduce morbidity and mortality rates in rural areas of poor developing countries. There is now a large stock of literature and project examples [both rural and urban], which demonstrate how this concept, commonly referred to as telemedicine, has been implemented for the benefit of poor populations in developing countries [1-4].

What we present is yet another use of ICT, in the telemedicine gamma of applications, aimed at reducing morbidity and mortality in the remote villages of Lundazi in the Eastern province of The Republic of Zambia.

Like most developing countries Zambia is characterized by an urban and rural demographic structure. The urban areas are relatively economically vibrant areas with access to basic infrastructure like telephones, electricity, treated water etc. The question in these areas is not the availability of basic infrastructure but rather the quality of service provided and the number of people able to make meaningful economic use of the infrastructure. The rural areas are more afflicted with actual lack of basic infrastructure needed to provide basic services [5]. This is usually due to distance from centrally located infrastructure in urban areas, rugged natural terrain that makes it difficult for service providers to economically set up the physical infrastructure and of course the economic viability of operations in terms of investment returns; the rural populations can not economically support the investments.

For the health sector this means lack of primary health care facilities and difficult access to referral centres due to bad or no roads, poor or no telecommunications and in most cases non-affordability of basic and referral health services. It also means lack of trained medical staff, who find it difficult to settle in these areas. At a national scale more than half of the Zambian trained medical staff have left the country for greener pastures abroad [6-8]. Such an exodus means that in Zambia, a country of 12 million people, there are only around 1200 registered doctors [9] and they are mainly based in urban areas. This situation is common throughout sub-Saharan Africa.

The 'The Virtual Doctor Project' in Zambia will use mobile clinics to facilitate free doctor-supported primary healthcare to remote, under-served areas of the Eastern province of Zambia and eventually to other rural and hard-to-reach areas of the country. The whole concept is modeled around the traditional 'store and forward' mode of telemedicine, where images and information are collected at remote health delivery sites and transmitted by email to doctors based in distant locations. This system will allow these 'virtual doctors' to offer on-going diagnostic assistance when required to clinical staff on the ground. The mobile clinic is planned to travel between a number of village sites in four-wheel drive vehicles specifically equipped with appropriate technologies for the purpose, setting up temporary clinics for several days at a time. The aim of the project is to reduce morbidity and mortality rates of easily treatable and preventable diseases in these rural areas. It also aims to reduce the number of referrals to distant referral hospitals, which are already struggling to cope with the high numbers of local patients seeking medical attention.

## Context

### The Project Area

**Figure 1:**
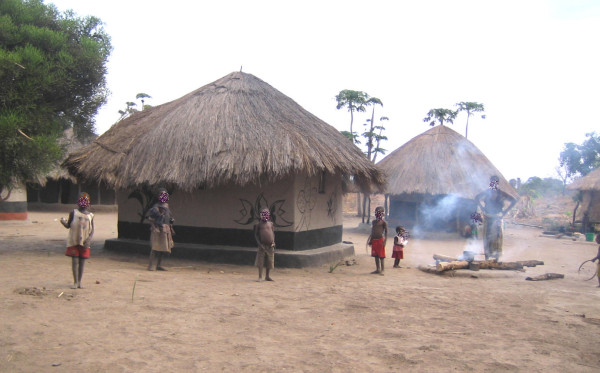
**Children in Lumimba Village, Lundazi District**.

Lundazi District is a rural area within the Eastern Province of Zambia. The project is targeting the remote hard-to-reach areas of the district including the 40,000 people living in Mwanya, Chitungulu and Kazembe which lie in the remote and western part of Lundazi along the Luangwa River (Figure 1). These are very isolated areas and often get cut off from the rest of the district during the rain season when the Luangwa river overflows. Most of the communities in this area are subsistence farmers, relying on harvests of maize, millet, rice, and pumpkins for their livelihood. They often experience drought in the dry season and floods during the rain season, which lead to crop failure and periods of hunger [10].

**Figure 2:**
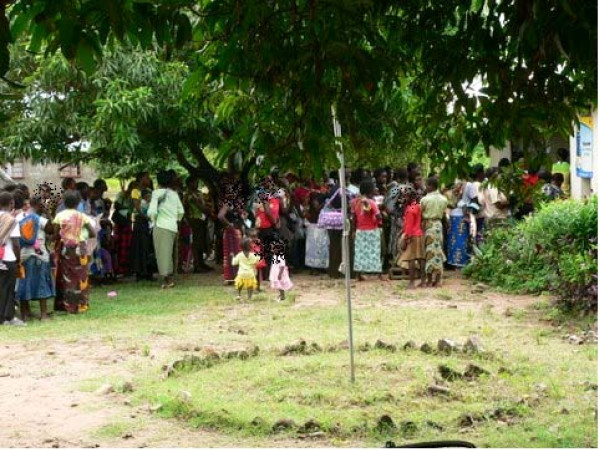
**Patients wait for registration outside Chitungulu Clinic, Lundazi District**.

**Figure 3:**
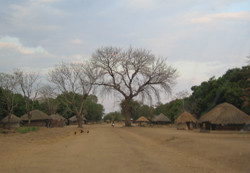
**The road through Chitungulu Village, Lundazi District**.

The most common ailments that have been recorded in the area are Malaria, Diarrhoea, Tuberculosis, HIV/AIDS, Respiratory Tract Infections, Maternity Complications, Animal and Snake Bites, Malnutrition and Anaemia. With such limited health care available, these complications often to lead to mortality [11]. The targeted area also has a critical shortage of trained medical staff. A key obstacle to patients gaining treatment is often the distances required to travel to local clinics or regional hospitals (Figure 2). This journey can often take several days by foot or by bicycle even for healthy people (Figure 3). This usually means a loss of income and with many health centers poorly staffed and under-equipped this effort may not guarantee any improvement for the patient. This means that often problems are not addressed until they become very serious. Early diagnosis and regular treatment would enhance the chances of recovery and reduce the number of referrals to hospital, helping to ease the burden on already over-stretched hospital services. A typical scenario that captures the desperation for health services in the Lundazi district is one reported by the World Medical Fund operating mobile clinics in neighboring Malawi: "*Typically the tarmac road would be over 25 km away and the nearest health facility a further 40 km. If the sick child made the journey, the choice would be between a government hospital where treatment, although free, is limited from chronic shortages of clinical staff and pharmaceuticals, or the mission sector where treatment is paid for, often beyond the means of the poor.......... The fact that the children's parents/guardians will travel for up to 2 days to be seen by our team indicates their lack of alternatives*".

### Telemedicine

Telemedicine is the use of ICTs for the delivery of healthcare. It has the potential to make a major contribution to improving access to health services while containing costs. Utilizing suitable technologies can enhance the quality and the reach of information and communication thus empowering impoverished communities. It will enable them to access health services previously beyond their reach. Influenced by wider information communication processes, telemedicine can respond to their health priorities and needs.

*'There is need to ensure that telemedicine and information communication technology [ICT] in the health sector reaches out to the poorest populations and that there is a strong focus on linking rural, remote, difficult environments that are underserved with the resources that are located in the central health services' *[12].

Examples of telemedicine abound and though applied differently in different places the objective remains the same: to provide otherwise expensive and unavailable healthcare to people living in remote under-serviced areas of poor countries.

Operation Village Health in Cambodia is a good example. Since 2001, Harvard-affiliated physicians have been providing clinical recommendations to Cambodian health workers caring for patients at a health centre in Rovieng and a referral hospital in Ban Lung. These consultations are based on text and image-rich clinical documents composed by Cambodian health workers, which are emailed to physicians in Boston and in Phnom Penh. Approximately 700 telemedicine-supported patient encounters have been completed. By the end of the second year of operation, average chief complaint duration decreased from 37 months at the project's inception to 8 months, a significant reduction. Referrals to hospitals outside of the village also decreased over the 28 month study period, due to improvements in local health care worker's skills and management techniques as well as the development of enhanced clinic capabilities that made it unnecessary for patients to travel to the distant hospital for certain basic tests. The number of patient transfers to facilities outside the village dropped by 51% per year since the establishment of the clinic [13].

Another example is the national telemedicine system in South Africa which was rolled out in 1999 using Integrated Services Digital Network [ISDN] at 256 kbps. The initial applications were tele-radiology, tele-ultrasound, tele-pathology and tele-ophthalmology^ii^. In 2003 the Ministry of Health in Zambia set up a National Telemedicine Steering Committee to study and implement telemedicine initiatives in the country. The committee sent a team to South Africa to study the telemedicine projects in that country and after the tour set up similar telemedicine projects around Zambia. The main objective of this initiative was to mitigate the shortage of skilled medical personnel in the country that was brought about by the massive emigration of personnel leaving the country with very few skilled medical specialists. This meant that the few specialists available had to be shared in the most efficient manner and telemedicine seemed to be a possible solution.

It is important to note that these initiatives were not meant to spread primary health care to the most remote areas of the country but rather sought to mitigate the shortage of specialist staff in the country. As such most telemedicine projects in Zambia are established around service delivery sites that already have the infrastructure and the capacity to utilize it. This is the major difference with the Virtual Doctor Project. Mobile clinics have been used a lot in Zambia to reach the rural populations for massive immunization campaigns and sometimes ante-natal and other primary health services. One such project is Longezia Mobile Medical Clinic^iii^, a mobile outfit operating in Sinazongwe in the Western province of the country. It was initially operated by two volunteer nurses under the CareNow Foundation since 2006, but was eventually handed over to the Mission Medic Air, Zambia [MMA]^iv^, which is also a charity based mobile medical service that uses light aircraft to reach remote rural areas of the country. MMA has been operating in the country since 1966 originally as backup medical service to mission hospitals. Since most mission hospitals now have resident doctors, it is now operated on a purely volunteer basis, where medical specialists donate their time and expertise to help people in remote areas of the country who otherwise would not have access to them. This flying doctors service highlights where the virtual doctor project would like to make a difference. Instead of being flown to remote locations around the country to render their special services, medical specialists can offer their time and expertise from the comfort of their own consulting rooms using modern communication technologies. The state of development of telemedicine in Africa [and around the world] however does not completely preclude occasional physical contact with a medical specialist. What is hoped for with the current state of technology and available resources is a reduction in the amount of this contact to a level where only the very necessary trips are made into the field and an increase in the number of consultations that may be completed in a given time period. Advanced communications systems will also enable a higher level of preparedness before a trip is made so that there are very few unknowns when the team gets there.

There has not been an initiative that has sought to combine mobility with specialist care at a distance as a main focus of the service. This is the special feature of the Virtual Doctor Project-the combination of mobility with telemedicine, making it possible for rural populations without telecommunications infrastructure and primary care facilities to both have access to primary and specialist care through the mobile clinics fitted with ICT equipment.

### Implementation

Bringing free medical expertise and treatment directly to under-served communities in hard-to-reach areas without incurring the expense of building clinics in every village and the difficulty of attracting doctors to live in these poor underserved rural areas is an ambitious objective. In trying to achieve this objective the Virtual Doctor Project will utilize mobile clinics, comprising an off-road vehicle staffed by local medical staff consisting of a medical assistant^v^, a midwife/nurse and a project officer. The project officer will be responsible for the logistical and technical aspects of the unit and driving the vehicle. These mobile units will travel between pre-designated sites and establish a temporary 'virtual doctor' clinic for several days, in a tent connected to the vehicle.

Using basic testing equipment and medical supplies brought by the unit, the clinic will offer a healthcare service performed by the staff, with support available for the more complex cases via telemedicine from a volunteer doctor (Virtual Doctor) in a remote location. In these cases the clinic staff will record a patient's symptoms and vital signs on a laptop, and then send the information by email with attached digital photographs and test results to the doctor based remotely, using a satellite link. Diagnostic advice will be returned by the following day and can then be used to either assist on-site treatment or to recommend referral to hospital.

Being a mobile clinic allows access to a far larger population than a static site, to the remote areas that most require a healthcare service, and to patients who might otherwise be unable or unwilling to travel the long distance to a clinic. In dealing with difficult cases, the clinic staff will be able to access advice and diagnostic/treatment assistance from remote specialists, either in Zambia or abroad, for back up diagnosis and dissemination of vital information. For patients that require referral to hospital the remote doctor will be able to contact the hospital directly, passing on vital information electronically. A fairly small roster of remote doctors can service many units in different areas, at far less expense than providing fully trained physicians at each site. The annual cost of each virtual doctor system including the equipment and satellite fees is approximately half the cost of a theoretical doctor's salary. Also, multiple opinions will be available from several different virtual doctors for each case.

Patient records will be kept electronically with the vehicle to ensure efficient on-going care, and can be easily forwarded to the remote doctor if required. A database of patient information will also allow the clinic to monitor its effectiveness and to contribute to local health statistics. As well as offering a basic healthcare service, the units will contribute to promote preventative solutions and education such as distributing mosquito nets and showing health education films to waiting patients. The project also intends to collaborate with other local projects that focus on key health issues such as HIV/Aids or maternal health to enhance the benefit for local communities.

It is envisaged that as the project develops more nursing staff or even trained paramedics will become part of the mobile clinic staff. Secondary vehicles to assist with transportation of patients referred to hospital will be incorporated to assist with referrals. More specialized equipment to offer better onsite testing such as x-ray machines and blood testing facilities to widen the scope of the service will also be incorporated.

### Telemedicine System

The equipment for the telemedicine email system is very portable and easy to use, and consists of a hard-wearing laptop and a satellite terminal unit that is similar in shape but smaller than a laptop and designed for that environment. Both emailing and VOIP [voice over internet protocol] calls are possible via RBGAN and Thuraya satellite networks. Electronic microscopes, vital-sign recorders, mobile digital x-ray units and other equipment are available that can be connected to the laptop to assist the clinical officers description of a complaint.

### Clinic Equipment

All of the required equipment for the clinic will be transported between locations in the back of the vehicle and then assembled on site. Particular attention will be paid to ensuring that the hygiene and cleanliness of the unit is maintained, and that the effect of sand, dust and dirt on the equipment is minimized. All sensitive equipment will be transported and stored overnight in protective flight cases in the vehicle [which will be securely locked when stationary], and the robustness and suitability to a harsh African environment will be a key factor in assessing the suitability of any item for use by the clinic. A small refrigerator will be fixed into the back of the vehicle and run permanently to protect medicines, and the rear of the vehicle will be carefully designed with multiple secure storage areas for general medicines and medical supplies. The clinic itself will comprise of a large tent attached to the rear of the vehicle with a collapsible bed, chairs and a table. The vehicle will return to the project workshop regularly, for general maintenance and repairs, and to restock with supplies.

### Partnership with the Zambian Government

As with many public services health is the responsibility of the sitting government to provide to its electorate. It is not the intention of the project to even pretend to take over this role from the government but rather to complement the efforts of the Zambian government in spreading primary health care to the neediest parts of the country. As such the project can not survive as a stand alone project but as an integral part of the existing health facilities in the country.

The objectives of the project fits snugly into the vision of the Health Reforms introduced in Zambia in 1999, which aimed to 'To *provide equity of access to cost effective quality health services as close to the family as possible in order to improve the health status of the people of Zambia' *The Ministry of Health [MoH] will therefore be the section leader in the project, and The Virtual Doctor Project will use MoH preferred health indicators in the existing Health Management Information System [HMIS] and the existing MoH district monitoring systems to monitor outcomes in a collaborative and constructive process.

Relevant stakeholders have already been consulted in the process of setting up the project including the district health offices in Lundazi as well as community leaders in Mwanya, Chitungulu and Kazembe.

### Declaration of Alma-Ata

The Virtual Doctor project's main purpose is to utilize technology to take medical expertise to remote communities in Africa, which in other words, means reshaping and improving primary health care for all. The Project promotes the right to health as a fundamental human right by supporting the Declaration of Alma-Ata, which was adopted at the International Conference on Primary Health Care, Kazakhstan in 1978. The Declaration urged the need to address priority health requirements and the fundamental parameters of health for all the people of the world, 'Health for All'. It highlighted the importance of primary health care and also verifies the WHO definition of health as a basic human right [14]. The Declaration broadened the medical model by acknowledging that civil society organizations are to have a pivotal role in shaping and improving health.

The concept was largely misunderstood and by 1994, a WHO review of world changes in health development since Alma-Ata despondently concluded that the goal of health for all by 2000 would not be met. Margaret Chan, the Director-General of WHO acknowledges that 'as we have seen, powerful interventions and the money to purchase them will not buy better health outcomes in the absence of efficient systems for delivery' [15].

Primary health care today is no longer so profoundly misinterpreted. Margaret Chan states that 'Primary health care increasingly looks like a smart way to get health development back on track'. Chan has pointed out that the most efficient and most effective way to organize a health system is related to the adoption of a primary health paradigm. She emphasizes that there is international evidence in relation to the fact that systems directed at primary health care provide for lower costs and better outcomes [16].

## Challenges

It is important to emphasize that telemedicine is not new to Zambia and while the Virtual Doctor Project is not necessarily introducing telemedicine or mobile clinics to the country, it is introducing a new and powerful combination of the two concepts targeted at alleviating the scarcity of primary health care facilities and specialist care in hard-to reach poor areas of the country. Whereas previous telemedicine projects have been concentrated in already developed and ready to go delivery sites, this project will endeavor to provide these services to communities where neither the infrastructure nor the health facilities exist. While these are noble and laudable objectives, a few challenges remain for the successful implementation of the project and the model laid out above may have to undergo radical changes dictated by practical and financial difficulties on the ground.

### Connectivity

The use of satellite connectivity as the major broadband platform is a familiar but specifically problematic option. Satellite bandwidth is expensive and many well intentioned telemedicine projects in Africa have failed to take off or gain traction specifically because of the high cost of satellite broadband access [17,18]. In most cases, as in the case of the Virtual Doctor project, the project area is not connected to the local telephone loop and does not yet have a mobile signal hence the choice of satellite capacity. The cost of the BGAN satellite connectivity targeted for this project is about $4,420.00 for one year email only at a capacity of 0.4 MB per email including a connection fee of $315.00^vi^. This drastically reduces the amount of imagery that can be sent over the link limiting the communication to predominantly text, which for telemedicine applications is hardly adequate. Needless to say this cost is not sustainable especially for a project dependent on charity funds. This connectivity challenge will have to be addressed in order to make meaningful use of the connectivity aspect of the project. It is hoped that by the time the pilot is done Lundazi district will have cellular mobile connectivity extending to the remote project areas. This will increase the broadband possibilities available and will provide some options for cheaper higher bandwidth connectivity.

### Equipment and vehicle

Transportation has always been an important aspect of health service delivery in Zambia. The problem has always been the maintenance and running costs for the vehicles. The road network in rural areas virtually ceases to exist and the make-shift routes that take its place are very temporal and seasonal. Many remote places are inaccessible in the rain season because of floods and this is the season when most rural inhabitants are prone to various waterborne diseases like cholera, malaria and diarrheal diseases. If swift and prompt action during the rain season is not possible because of bad roads, then serious doubts arise as to the efficacy and suitability of the mobile outfit.

### Political Climate

In June 2009 controversy broke out over a decision by the government to buy nine mobile clinics from China at a cost of $53 million dollars. The public wanted to know whether this was the best way to utilize national resources as the money could be used to build several rural health centers and provide housing for rural health staff among other things^vii^. Justification for the purchase was sought in the fact that the resources to be employed were actually in aid form and could therefore not be diverted to other uses. The public felt that this was definitely not the best form of investment in the people's health and a heated debate ensued. This brings out the reality that although these services exist in project form around the country, a full government investment will take a little more convincing before serious funds can be allocated. On the other hand mobile health services cannot forever be provided by projects funded by charity. The sustainability of these projects lies in their eventual transition into the main health delivery system of the country.

Without the political support of the people through their representatives and the larger awareness of the benefits of these projects on a national scale, there is little hope that this transition will ever take place. It is for this reason therefore that the existence and benefits of mobile health clinics in the country should be publicized a lot more through the national media and through lobby groups to parliament representatives so that eventually they find their place in the main health delivery system of the country. It would be a shame if the good work done by the Mission Medic Air, Longezia Mobile Medical Clinic, the Virtual Doctors project and many other mobile health projects, that are honestly trying to fill the gap, remain outside the main health care system of the country. This would condemn them to perpetual dependence on charity which can not be guaranteed. The implication of this is the sad cessation of a vital health service to poor people when the charity funds run out.

It is clear that the resistance to the idea of the purchase of mobile health clinics was mainly based on lack of information on what benefits similar projects have actually brought to the rural poor in the country. This lack of knowledge and the precedence set by the public outcry against the purchase of the mobile clinics will have to be addressed by concrete demonstration of positive results from similar projects in the country.

## Concluding Remarks

In this commentary we have introduced an enhanced telemedicine project which is yet to be implemented in Lundazi district in Eastern Zambia. We have discussed other similar projects in other countries and seen how the combination of mobility and connectivity may enhance and increase access to specialist medical care in remote areas of the country.

This is the introductory description of what is yet to be done by the Virtual Doctor project, which will be followed later by a result oriented report of what will actually happen in the field during implementation. The aim is to provide concrete information that may lend credence to future plans by the government to include these modern approaches to expanding health care to remote areas of the country.

The issues of sustainability are very closely related to the success or failure of explicitly demonstrating these positive outcomes. This is because sustainability of projects like this lies in their adoption and expansion by the government of the day and a dramatic downscaling of reliance on charity donations. It is unethical and unacceptable that provision of health care to rural needy populations in Africa should be dependent on charity donations. If there are technological advances that can stretch the reach of primary medical care to these areas, as we posit in this write-up, then it should be imperative on the government of the day to adopt these technologies without regard to the market forces that usually work to impede such adoption. This sustainability is both ethically and technically vital in order to afford continued provision of telemedicine as an instrumental resource for the fundamental good of health in rural Africa (c.f. [19,20] and [21]).

## Competing interests

The authors declare that they have no competing interests.

## Authors' contributions

All the named authors have read and approved the final manuscript. All authors contributed equally to the writing of this paper.

## About the Authors

Evans N Mupela

Evans Mupela is a PhD graduate of the Economics and Policy Studies of Technical Change program at UNU-MERIT in Maastricht. He is based at Walter Sisulu University of Technology in the Eastern Cape of South Africa, where he lectures in Computer Science. His main research interest is in the effects of ICTs on African development.

Paul Mustarde

Paul, has many years of Project Management and organizational management experience in the UK, both in the environmental-sustainable development sector of the construction industry and the music industry. Paul lives in the South of England running the UK based operations for the Virtual Doctor Project

Huw Jones

Huw spent 15 years in the safari industry in Africa, leading safaris and running lodges in Eastern Zambia and Tanzania. He returned to the UK to complete a Masters in Environment and International Development, and has subsequently worked for the European Union and United Nations specializing in sustainable development. He is currently working on a large scale community agriculture development project in Southern Zambia.

## Footnotes

^i ^The term communication technologies is used here as a generic reference to all sorts of electronic communication modes that include telephones [mobile and fixed],radios, internet, television etc

^ii ^See article by S.Gulube, and S Wynchank Telemedicine in South Africa: Success or Failure? Medical Research Council, Pretoria, South Africa. J Telemed Telecare. 2001;7 Suppl 2:47-9.

^iii ^more information on http://www.gchope.org/longezia-mobile-medical-clinic.html

^iv ^http://www.mma-zambia.org/page64.html

^v ^A Medical Assistant in Zambia is the equivalent of a mid-level medical practitioner/provider in America or Western Europe.

^vi ^The full project budget including these figures is available from the project document page 6 accessible at http://www.virtualdoctors.org

^vii ^See The Zambian Economist on http://www.zambian-economist.com/2009/07/foreign-aid-for-mobileclinics.html
